# The Effect of Type 2 Diabetes on Bone Quality: A Systematic Review and Meta-Analysis of Cohort Studies

**DOI:** 10.3390/ijerph22060910

**Published:** 2025-06-06

**Authors:** Omorogieva Ojo, Yemi Onilude, Joanne Brooke, Victoria Apau, Ivy Kazangarare, Osarhumwese Ojo

**Affiliations:** 1 School of Health Sciences, Faculty of Education, Health and Human Sciences, University of Greenwich, Avery Hill Campus, London SE9 2UG, UK; o.onilude@greenwich.ac.uk (Y.O.); v.apau@greenwich.ac.uk (V.A.); i.kazangarare@greenwich.ac.uk (I.K.); 2 Centre of Social Care, Health, and Related Research, Birmingham City University, Westbourne Rd., Birmingham B15 3TN, UK; joanne.brooke@bcu.ac.uk; 3 South London and Maudsley NHS Foundation Trust, London SE13 6LH, UK; osarhumwese.ojo@slam.nhs.uk

**Keywords:** type 2 diabetes, bone quality, bone mineral density, trabecular microarchitecture, fracture risk, trabecular bone score

## Abstract

Background: There is a significant knowledge gap and limited studies have been carried out to evaluate the effect of type 2 diabetes (T2D) on bone quality and skeletal fragility. Previous reviews have tended to focus primarily on bone mineral density (BMD) as a measure of bone quality. However, BMD does not fully reflect the risk of fracture, cannot distinguish between cortical and trabecular bone, and bone fragility in patients with T2D results not only from alterations in bone mineralisation, but also due to changes in bone microarchitecture. In this regard, assessment tools such as trabecular bone score (TBS) and trabecular microarchitectural parameters could be useful and practical tools for examining bone status in people with T2D. Aim: This review aims to examine the effect of type 2 diabetes on bone quality based on a variety of assessment tools. Method: The PRISMA checklist and PICOS framework were relied on for this systematic review and meta-analysis. Two researchers conducted the searches from database inception until 24/02/25. Databases including Academic Search Premier, APA PsycArticles, APA PsycInfo, CINAHL Plus with Full Text, MEDLINE, and the Psychology & Behavioral Sciences Collection were searched for relevant articles. The reference lists of articles were also searched. The Review Manager 5.4.1 software was used to carry out the meta-analysis. Results: Ten studies were included in the systematic review, while nine studies were included in the meta-analysis. Based on the narrative synthesis and meta-analysis, four distinct themes were established: bone mineral density, TBS and trabecular microarchitectural parameters, fracture risk, and body mass index (BMI). The meta-analysis of the effect of T2D on BMD showed that T2D significantly (*p* < 0.05) increased lumbar spine, total hip, femoral neck, and narrow neck BMD compared with controls. The mean differences (MDs) for the respective parameters were 0.04 (95% CI, 0.03, 0.05, *p* < 0.0001); 0.05 (95% CI, 0.02, 0.08, *p* = 0.002); 0.07 (95% CI, 0.04, 0.10, *p* < 0.0001); and 0.03 (95% CI, 0.01, 0.05, *p* = 0.0005). While there was a significant reduction (*p* < 0.0001) in the patients with T2D with respect to volumetric BMD, involving two studies and 1037 participants, with an MD of −12.36 (95% CI,−18.15, −6.57, *p* < 0.0001), T2D did not appear to have a significant effect (*p* > 0.05) on total BMD and area BMD compared to controls. In relation to TBS and trabecular microarchitectural parameters, the effect of T2D was not significant (*p* > 0.05) compared with controls. Furthermore, T2D did not have a significant effect (*p* > 0.05) on the incidence of hip fracture and non-spine fracture compared to controls. Following meta-analysis, it was found that the T2D significantly (*p* < 0.05) increased BMI compared to controls with an MD of 0.94 (95% CI, 0.74, 1.14, *p* < 0.0001). Conclusions: Type 2 diabetes significantly increased (*p* < 0.05) lumbar spine, total hip, femoral neck, narrow neck BMD, and body mass index compared with controls. However, type 2 diabetes did not appear to have a significant effect (*p* > 0.05) on TBS, trabecular microarchitectural parameters, and the incidence of hip and non-spine fracture.

## 1. Introduction

The prevalence of type 2 diabetes is on the increase globally, partly due to the adoption of a Western lifestyle and other environmental influences [1]. People who develop type 2 diabetes (T2D) are also at risk of developing complications including acute and chronic complications such as bone-related problems [2,3,4]. These diabetic problems have implications for the patients in terms of the cost of treatment, quality of life, and the risk of mortality [5]. What has become evident is that most research studies conducted to examine the acute and chronic conditions in people with T2D have tended to focus on hyperglycaemia, diabetic ketoacidosis, hyperosmolar hyperglycaemic state, neuropathy, nephropathy, and retinopathy [2]. However, T2D has also been shown to affect bone metabolism and increase the risk of bone-related complications, known as diabetic osteopathy, that may result from impaired cortical and trabecular microarchitectural parameters, despite preserved bone mineral density (BMD) [4,6].

Description of Bone Quality

Bone quality involves the geometric and material factors that contribute to fracture resistance [7]. On the other hand, bone strength encompasses both bone quantity and quality [7]. As BMD has limitations in predicting fracture risk, especially in people with T2D, scientific and clinical interests are now focused on other measures of bone quality that could improve fracture risk prediction [7]. The bone geometric parameters comprise the macroscopic geometry of the whole bone and the microscopic architecture of the trabeculae [7]. The material factors involve the material properties of the constituent tissue that are drawn from the composition of the primary microstructural constituents, collagen and minerals [7]. In relation to bone tissue, it can be broadly divided into two types: cortical bone (compact bone or dense bone) and trabecular bone (cancellous or spongy bone) [8]. These distinctions between the two types of bone tissues are based mainly on porosity [8]. The role of diabetes in the pathogenesis of bone fragility may be due to its effect in suppressing bone remodelling, including the impairment of the healing of microfractures in mechanically loaded bones which may predispose individuals with diabetes to fractures [9]. In particular, it has been reported that chronic hyperglycaemia and the related advanced glycation end products have been implicated in the process of increased bone fragility in people with type 2 diabetes [9]. Furthermore, chronic inflammation and oxidative stress could significantly affect osteogenesis and increase bone resorption [9].

Why It Is Important to Do This Review

It would appear that there is a significant knowledge gap and limited studies that have been carried out in order to evaluate the effect of type 2 diabetes on bone quality and skeletal fragility [10,11]. For example, Ma et al. [12] conducted a meta-analysis of observational studies to assess the association between BMD and type 2 diabetes. However, it has been reported that BMD and the World Health Organisation Fracture Risk Assessment Tool (FRAX) underestimate fracture risk in people with diabetes as the metabolic contributors to bone are complex and multifactorial [13,14,15]. Bone cells, structure, vasculature, bone quality, and fracture risk may be influenced by hyperglycaemia, hyperinsulinaemia, diabetes duration, and glucose management [15]. BMD does not adequately reflect the tendency of patients with T2D to develop bone fragility [15,16]. BMD does not fully reflect the risk of fracture, cannot distinguish between cortical and trabecular bone, and the information it provides on bone quality is limited [17]. Furthermore, bone fragility in patients with T2D results not only from alterations in bone mineralisation, but also due to changes in bone microarchitecture [17]. In this regard, the trabecular bone score (TBS) which indicates a reduced number of trabeculae, less connectivity, and impaired bone microarchitecture is one of the most practical tools for examining bone status in people with T2D [17].

Therefore, it will be useful to evaluate bone quality as an indication of the risk of skeletal fragility in people with type 2 diabetes using a variety of bone quality measurement tools.

Aim: The current review aims to examine the effect of type 2 diabetes on bone quality based on a variety of assessment tools.

Research Question: What are the effects of type 2 diabetes on bone quality?

## 2. Method

The Preferred Reporting Items for Systematic Reviews and Meta-Analyses (PRISMA—Supplementary Table S1) [18] was relied on for this review. The protocol for the systematic review and meta-analysis was registered with PROSPERO (Registration Number: CRD420250654178).

Participants of Interest: People with T2D were the population of interest.

Outcome Measures: These included BMD, TBS, and trabecular microarchitectural parameters, fracture risk assessment, and body mass index (BMI).

Search Strategy

Searches were carried out in EBSCOHost and the databases included Academic Search Premier, APA PsycArticles, APA PsycInfo, CINAHL Plus with Full Text, MEDLINE, and the Psychology & Behavioral Sciences Collection, which were searched for relevant articles. The reference lists of articles were also searched. The search terms and the research question were based on the Population, Intervention, Comparator, Outcomes and Study (PICOS) framework. The searches were carried out from database inception until 24/02/25 and involved two researchers (OO and OO); one carrying out the initial search and the other repeating the process to confirm the result of the searches. The search terms are outlined in Table 1, and these were combined using Boolean operators (AND/OR). The duplicates were removed in EndNote (Analytics, Philadelphia, PA, USA).

Collection of Data

**Figure 1:**
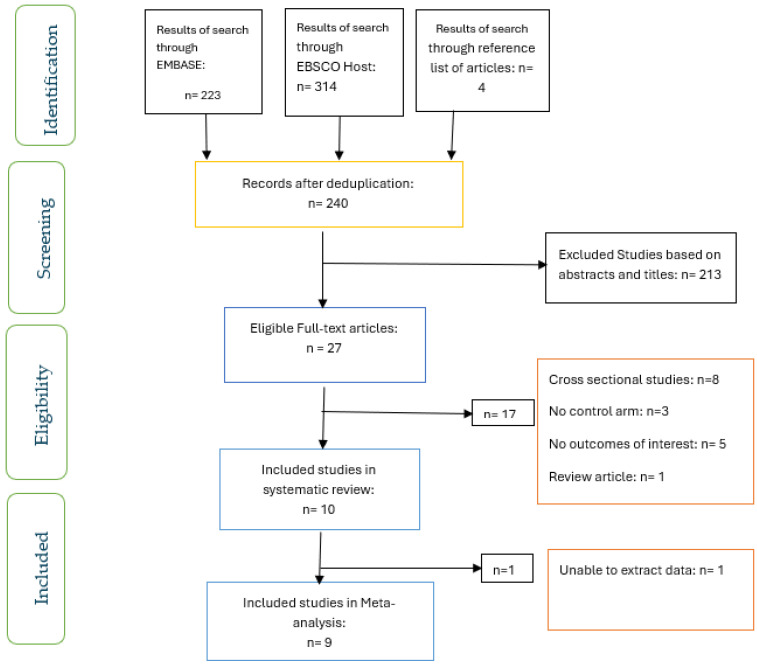
PRISMA flow chart of studies included.

The screening of articles for eligibility and inclusion was carried out by two researchers (OO and OO) who worked independently (Figure 1). Differences between the researchers were resolved through discussion.

Study Selection

Inclusion Criteria: Cohort studies and patients with type 2 diabetes were included in the review. In addition, articles written in English were included.

Exclusion Criteria: Patients with type 1 diabetes, gestational diabetes, and articles not written in English were excluded from the review. Animal and in vivo studies were also excluded from the review.

Data Extraction and Management

Two researchers (YO and OO) extracted the qualitative data from the studies included in the review and three researchers (VA, IK, and YO) extracted the quantitative data for meta-analysis and this was cross-checked by a fourth researcher (OO). The characteristics of the studies included such as citation, the research method, the aim of the study, the mean age, the sample size, and outcomes were extracted as part of the qualitative data. The narrative synthesis of the findings of the articles included was also conducted.

Quality Assessment of Studies.

The studies included were evaluated for quality using the Critical Appraisal Skills Programme (CASP) checklist for cohort study [19]. Two researchers (OO and VA) worked independently to assess the risk of bias/quality of the included studies.

Meta-Analysis

The Review Manager 5.4.1 software was used to carry out the meta-analysis [20]. The measure of heterogeneity relied on the use of the *I*^2^ statistic [21], and *p* < 0.10 was the statistical significance of heterogeneity. The sensitivity analysis involved the removal of one study at a time from the meta-analysis. The means ± SEM and confidence intervals reported in some studies were converted to means ± SD for the meta-analysis.

Some of the studies [22,23,24] reported results separately for male and female participants. Therefore, those findings were analysed separately and subgroup analysis was carried out for these studies.

Measures of Effects: The fixed effects model and mean difference were used for the meta-analysis, except in the analysis of trabecular number, when the standardised mean difference was used.

Effect Size

Forest plots were used to present the meta-analysis and *p* < 0.05 was used to assess the overall effect of the intervention and the level of statistical significance.

## 3. Results

Figure 1 shows the screening for eligibility of the ten studies that were included in the systematic review, and the nine studies included in the meta-analysis. The characteristics of the studies included are shown in Table 2. Two studies each were conducted in the USA [25,26] and Japan [23,27], while one study each was carried out in the UK [22], the United Arab Emirates [28], the Netherlands [16], Canada [29], China [24], and Denmark [30]. All of the participants had T2D and were compared with those without diabetes.

The Evaluation of the Quality/Risk of Bias of the Studies Included

All the studies included in this systematic review and meta-analysis effectively addressed most of the questions on the CASP [19] checklist, including whether the study addressed a clearly focused issue, if the outcomes were accurately measured to minimise bias, whether the results could be applied to the local population, and if the results of the study fit with other available evidence. However, although data in the Van Hulten et al. [30] study appeared to have been collected in an acceptable way, we could not find any statement relating to the ethical approval of the study. Furthermore, the Jawhar et al. [28] study did not appear to have clearly identified all important confounding factors. The potential publication bias of the included studies was assessed by examining whether the results reported were due to reporting bias. Based on the evaluation of the included studies, it was clear that there was no publication bias as the authors of this review believed in all the results of the studies.

Information regarding potential confounding variables (duration of diabetes, glycaemic control measures, and other medications) captured in the studies included are presented in Table 3.

Based on the narrative synthesis and meta-analysis, four distinct themes were established: bone mineral density, trabecular bone score and Microarchitectural parameters, fracture risk, and body mass index.

Bone Mineral Density (BMD)

In the recently diagnosed diabetic subjects in Dennison et al.’s [22] study, BMD was identified as higher with stronger relationships in women (*p* < 0.001) than men (*p* < 0.05). These findings were weakened by BMI adjustments. In addition, the BMD was notably higher (unadjusted and adjusted for lifestyle and BMI) in men who had a recent diagnosis of diabetes. Between the overall femur and femoral neck BMD, positive links were identified with insulin resistance measures (r = 0.17–0.22) in both men and women.

Oei et al.’s [16] findings concluded that the subjects with diabetes were older and had a higher BMI. They also had increased serum insulin levels, as well as raised creatinine levels, and were taking diuretics more regularly than the non-diabetes group [16]. A higher BMD, thicker cortices, and narrower femoral necks were seen in the inadequately controlled diabetes (ICD) group compared to the adequately controlled diabetes (ACD) and no diabetes (ND) groups, respectively [16]. In T2D, poor glycaemic control was linked to a higher BMD, as were thicker femoral cortices in bones that are narrower [16].

Wang et al. [24] identified that both men and women with diabetes were significantly older (*p* < 0.001). In addition, both sexes who had a diagnosis of diabetes had a lower volumetric bone mineral density (vBMD) compared to the non-diabetic subjects [24]. However, the vBMD figures were non-significant after they were adjusted according to age. In addition, after adjusting the findings for age in the dual-energy X-ray absorptiometry (DXA) sub-cohort, the results showed a significant rise in areal bone mineral density (aBMD) in men with diabetes [24].

In Heilmeier et al.’s [26] study, a significant decrease in the total BMD was noted in the control group (−3.8%, Tt.BMD, *p* = 0.001) and cortical area (Ct.Ar) (−3.9% Ct.Ar, *p* = 0.007) with a significant increase in the cortical pore diameter (control: +8.8% Ct.Po.Dm, *p* = 0.007) [26]. Both patients with T2D and control participants similarly demonstrated significant losses in cortical BMD (controls: −5.7% Ct.BMD, *p* < 0.001, T2D subjects: −3.9% Ct.BMD, *p*= 0.003) [26]. For women with diabetes in Pritchard et al.’s [29] study, lumbar spine was greater (*p* < 0.05) compared to those without diabetes at both the baseline and follow-up points [29].

In Jawhar et al.’s [28] study, the prevalence of osteoporosis was notably higher in the patients in the diabetes group (*p* ≤ 0.01) with the Z-score lumbar spine values, L1 and L3, also significantly higher (*p* ≤ 0.05) than the control group. In the diabetic and control groups, BMD and T-score values were identical. There were notably higher values of BMD, T-score, and Z-score in the left femur total hip in younger (age range of 40–49 years) diabetic patients (*p* ≤ 0.05) [28]. In the L3 region of the spine, patients with diabetes in the age range of 50–59 years had a very noticeably higher BMD value and Z-score [28].

Iki et al. [27] reported a significantly higher weight and areal BMD (aBMD) observed in men with T2DM in comparison with those without T2DM. Independently of bone turnover and pentosidine levels, hyperglycaemia and increased insulin resistance were seen to be associated with a low TBS [27]. Mitama et al. [23] found spine BMD to be significantly increased (*p* < 0.05) in men and women with diabetes.

**Figure 2:**
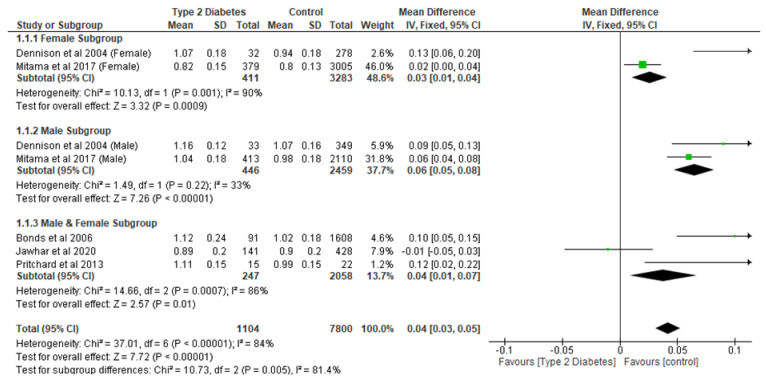
The effect of type 2 diabetes on lumbar spine bone mineral density (g/cm^2^) [22,23,25,28,29].

**Figure 3:**
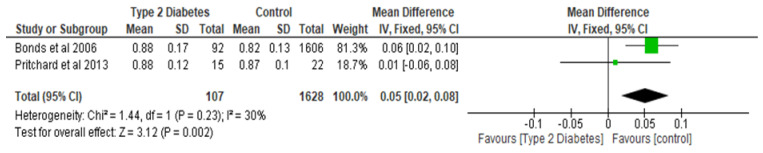
The effect of type 2 diabetes on total hip bone mineral density (g/cm^2^) [25,29].

**Figure 4:**
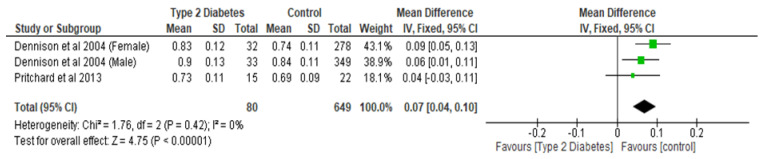
The effect of type 2 diabetes on femoral neck bone mineral density (g/cm^2^) [22,29].

**Figure 5:**
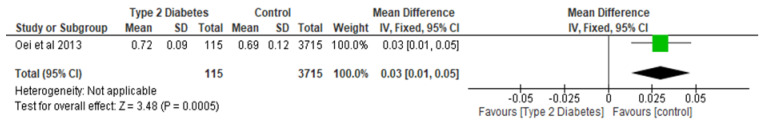
The effect of type 2 diabetes on narrow neck BMD (g/cm^2^) [16].

The meta-analysis of the effect of T2D on BMD showed that T2D significantly (*p* < 0.05) increased lumbar spine, total hip, femoral neck, and narrow neck BMD compared with controls (Figure 2, Figure 3, Figure 4 and Figure 5). With respect to lumbar spine BMD, seven studies involving 8904 participants were analysed and the mean difference (MD) was 0.04 (95% CI, 0.03, 0.05, *p* < 0.0001) (Figure 2). The subgroup analysis still showed that T2D significantly increased (*p* < 0.05) lumbar spine BMD in the different subgroups.

Total hip BMD involved two studies and 1735 participants in the analysis, and the MD was 0.05 (95% CI,0.02, 0.08, *p* = 0.002) (Figure 3), while the femoral neck BMD had three studies and 729 participants in the analysis, with an MD of 0.07 (95% CI, 0.04, 0.10 *p* < 0.0001) (Figure 4). The analysis of the narrow neck BMD involved one study and 3830 subjects, with an MD of 0.03 (95% CI, 0.01, 0.05, *p* = 0.0005) (Figure 5). Following the sensitivity analysis, the results with respect to lumbar spine and femoral neck remained consistent (*p* < 0.05). However, the result was not significant (*p* > 0.05) when the Bonds et al. [25] study was removed from the analysis, with respect to the total hip BMD.

**Figure 6:**
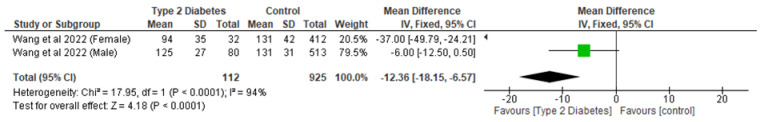
The effect of type 2 diabetes on volumetric bone mineral density [24].

While there was a significant reduction (*p* < 0.0001) in the patients with T2D group with respect to volumetric BMD, involving two studies and 1037 participants, with an MD of −12.36 (95% CI,−18.15, −6.57, *p* < 0.0001) (Figure 6), T2D did not appear to have a significant effect (*p* > 0.05) on total BMD and areal BMD compared to controls (Table 4).

Trabecular Microarchitectural Parameters

Heilmeier et al. [26] reported significant changes in most trabecular microarchitectural parameters for T2D- postmenopausal women without a history of fragility fractures and non-diabetic postmenopausal female control groups. For both T2D and controls, there was a significant decrease in trabecular number followed by a significant increase in trabecular thickness and trabecular spacing (controls: +8.8% trabecular thickness, *p* = 0.024, + 7.5% trabecular separation, *p* = 0.017; DM: +8.4% trabecular thickness, *p* = 0.039, 6.0% trabecular separation, *p* = 0.032) [26]. With regard to the ultradistal tibia, annualised percentage changes in the density parameters (total BMD, trabecular BMD, and cortical BMD) and in the cortical bone parameters, including cortical porosity, cortical pore volume, and cortical tissue mineral density, were similar between all three groups (*p* > 0.05) [26].

There was a higher percentage increase in the number of trabecular bone holes after adjusting for ethnicity in comparison to the controls [29]. However, after adjustment for multiple comparisons there was no significance (*p* = 0.090). Between the groups, there were no differences in the change in other bone microarchitecture variables [29]. Iki et al.’s [27] study did not identify measurable differences in TBS and the frequency of past osteoporotic fractures between the T2D group and control group.

**Figure 7:**
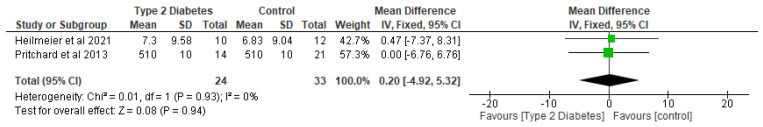
The effect of type 2 diabetes on trabecular thickness (µm) [26,29].

**Figure 8:**
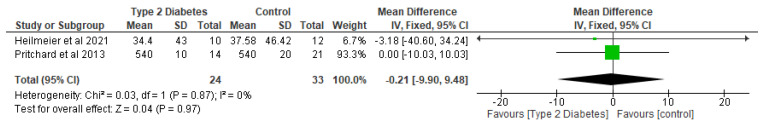
The effect of type 2 diabetes on trabecular separation (µm) [26,29].

**Figure 9:**
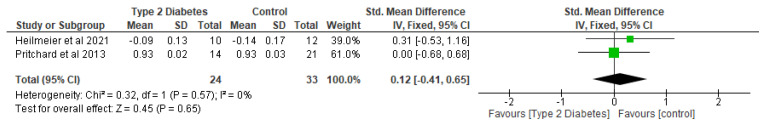
The effect of type 2 diabetes on trabecular number (standardised mean difference) [26,29].

In relation to trabecular microarchitectural parameters, the meta-analysis of the effect of T2D was not significant (*p* > 0.05) with respect to trabecular thickness, trabecular separation, trabecular number, and trabecular BMD (Figure 7, Figure 8 and Figure 9; Table 4). After the sensitivity analysis, the results with respect to trabecular thickness, trabecular separation, and trabecular number remained consistent (*p* > 0.05).

Furthermore, the effect of T2D on hole size, the number of holes, and bone volume fraction were also not significant (*p* > 0.05) compared with the control group (Table 4).

Fracture Risk

Bonds et al. [25] identified a significantly increased risk of any fracture in women with diabetes compared with non-diabetic women (*p* < 0.0001) after follow-up for 7 years. Women with diabetes had a higher fracture rate, with fracture by location identified at multiple sites, such as the hip, pelvis, upper leg; lower leg, ankle, knee; foot; upper arm shoulder, elbow; and spine and tailbone (*p* < 0.0001) [25]. The results also showed an equal rate of fractures to the lower arm, wrist, and hand in both groups [25]. However, among black women with diabetes, a higher risk of fracture at multiple sites such as the hip, pelvis, upper leg; foot; spine and tailbone was also reported (RR 1.33, 95% CI 1.00 –1.75) in comparison to non-Hispanic white (NHW) diabetic women (RR 1.18, 95% CI 1.08–1.29) [25].

Among the Japanese cohort, Mitama et al. [23] found a significantly higher risk of fracture among T2D men and women with high C-reactive protein (CRP) compared with the non-diabetes group with a low CRP (in men, hazard ratio [HR] 1.47, 95% CI: 1.02–1.98; in women HR 1.41, 95% CI: 1.04–1.92). Likewise, after age, BMD, and previous fractures adjustments, CRP was associated with a higher fracture risk in both sexes (in men, HR 1.04, 95% CI: 1.003–1.06; in women HR 1.07, 95% CI: 1.03–1.13) [23]. Oei et al. [16] also reported an increased fracture risk in participants with ICD compared to non-diabetic individuals.

In women, crude incidence rates (IRs) for hip fractures, non-spine fractures, and major osteoporotic fracture (MOF) were not significantly different (age > 30) in patients with T2D that are not using insulin (IR hip: 8.7; 95% CI 6.8–11.0) or using insulin (IR hip: 11.1; 95% CI 6.8–18.0) compared with women without T2D (IR hip: 7.0; 95% CI 6.6–7.4) [30]. Also, in men with T2D not taking insulin, IRs were not significantly different (IR hip: 4.6; 95% CI 2.6–8.0), compared to those using insulin (IR hip: 11.5; 95% CI 6.2–21.4) and men without T2D (IR hip: 6.3; 95% CI 5.5–7.1) for all three fracture types [30].

**Figure 10:**
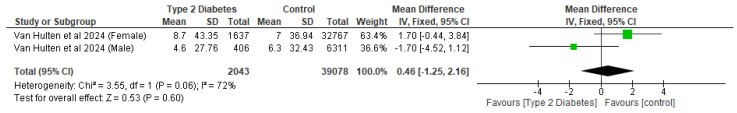
The effect of type 2 diabetes on the incidence of hip fracture (Incidence Rate) (1000 PYs—Person Years) [30].

The meta-analysis of the risk of fracture showed that type 2 diabetes did not have a significant effect (*p* > 0.05) on the incidence of hip fracture and non-spine fracture compared to controls (Figure 10, Table 4). With respect to the incidence of hip fracture, two studies involving 41,121 participants were analysed and the MD was 0.46 (95% CI, −1.25, 2.16, *p* = 0.60) (Figure 10). After the sensitivity analysis, the results continued to show that the difference between the two groups was not significant (*p* > 0.05).

Body Mass Index

Wang et al. [24] noted that both men and women with diabetes were much older (*p* < 0.001), and their BMI was higher than that of women who did not have diabetes. Similarly, Oei et al. [16] showed that those with diabetes were older and had a higher BMI and higher serum insulin and creatinine levels. Oei et al. [16] also noted that these subjects used diuretics more regularly than the non-diabetes group.

**Figure 11:**
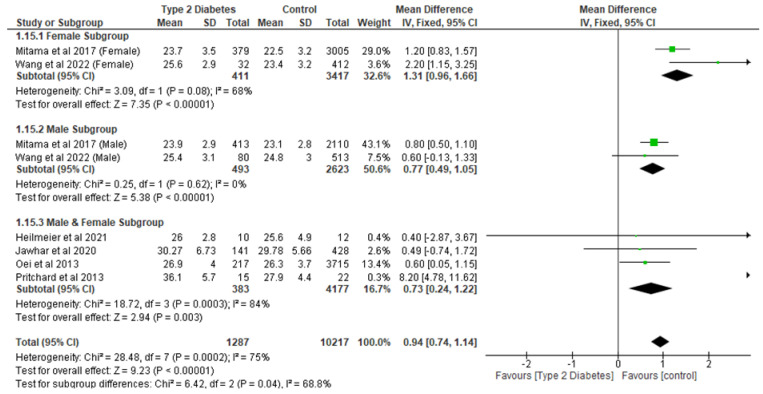
The effect of type 2 diabetes on body mass index (kg/m^2^) [16,23,24,26,28,29].

Following the meta-analysis, it was found that the T2D group had a significantly (*p* < 0.05) increased BMI compared to controls. The analysis involved eight studies and 11,504 participants, with an MD of 0.94 (95% CI,0.74, 1.14, *p* < 0.0001) (Figure 11). Following the sensitivity analysis, the effect of T2D on BMI compared with controls remained significantly different (*p* < 0.05). The subgroup analysis showed that T2D significantly increased (*p* < 0.05) BMI in the different subgroups.

## 4. Discussion

The results of this systematic review and meta-analysis show that T2D significantly increases (*p* < 0.05) lumbar spine, total hip, femoral neck, narrow neck BMD, and BMI compared with controls. However, T2D did not appear to have a significant effect (*p* > 0.05) on total BMD, areal BMD, TBS, trabecular microarchitectural parameters, and the incidence of hip and non-spine fracture compared to controls.

The findings of this review with respect to most of the parameters are in line with the results of previous primary research studies and reviews. For example, the meta-analysis of observational studies conducted by Ma et al. [12] found that patients with diabetes had a significantly higher (*p* < 0.05) BMD at the femoral neck, hip, and spine compared with those without diabetes. Furthermore, Sosa et al. [31] noted that, while studies that are small-scale may have reported either unchanged or decreased BMD in people with diabetes, all large epidemiological studies have now unanimously identified an increase in bone mass [31]. The clinical relevance of increased BMD in diabetes is that a high BMD in people with inadequately controlled diabetes may be a reflection of skeletal complications of the disease. Therefore, the use of BMD alone may not be adequate to predict or diagnose the risk of fracture in these people [16].

Sihota et al. [32] did not find significant differences in areal BMD between the groups, which was also confirmed in the result of the current review. However, in contrast to our results, Sosa et al. [31] did not find significant differences between patients with diabetes and controls with respect to femoral neck BMD, and suggested the differences reported in other studies may be due to differences in the weight of the patients between those with diabetes and controls, a well-known determinant of femoral bone density.

The mechanism of how diabetes influences bone quality including bone mineral density and trabecular microarchitectural parameters remains unclear and it is an evolving area of research [33]. However, several pathways, such as obesity, hyperglycaemia, hyperinsulinemia, growth factor deficiency, and neuropathy, have been proposed as causes of abnormal bone physiology in patients with diabetes [12,31,33]. The mechanism through which obesity increases BMD may be through the release of a broad range of adipokines from the adipose tissue, and these adipokines have been implicated either directly or indirectly in the regulation of bone remodelling [12]. For example, leptin has been found to be higher in men with diabetes compared with control, and that leptin may induce bone growth by stimulating osteoblast proliferation and differentiation in vitro, and inhibiting osteoclastogenesis [12].

With respect to the role of hyperinsulinaemia in influencing increased BMD, it has been shown that insulin resistance and excess insulin are common features in people with type 2 diabetes, and that insulin has an anabolic effect on bone due to its structural homology to Insulin-like Growth Factor-1 (IGF-1) by interacting with the IGF-1 receptor which is present on osteoblasts [12]. Based on this, it has been suggested that hyperinsulinaemia may have a mitogenic effect on osteoblasts and their differentiation by stimulating the IGF-1 signalling pathway [12].

According to La Fontaine et al. [33], a possible mechanism by which neuropathy affects bone turnover may be via the neuropeptide calcitonin gene-related peptide, that has been reported to be downregulated in patients with neuropathy. In women with T2D, increased androgen levels have been implicated in the alteration of bone quality [31].

In the present review, BMI was significantly (*p* < 0.05) increased in patients with T2D compared with controls. People with a high BMI are often associated with higher body fat content, which could be converted into fat-related hormones [34]. There is evidence body fatness may have an effect on the accuracy of dual-energy X-ray absorptiometry (DXA)-based BMD measurements in obese patients with diabetes [12].

Chronic hyperglycaemia has also been implicated as one of the pathways through which diabetes may impact bone quality [34]. In patients with T2D, prolonged disease duration may lead to a gradual decline in insulin production and function, and long-term insulin deficiency can lead to chronic hyperglycaemia and decreased bone turnover, affecting osteoclast activity and promoting bone resorption [34].

Chronic hyperglycaemia has also been reported to affect osteoblast function, leading to decreased bone formation and mineralization [21,29]. In addition, advanced glycation end-products (AGEs) and bone turnover are intermediate factors which play a considerable role in the association between hyperglycaemia and impaired bone microarchitecture [27].

The study by Sihota et al. [32] provides evidence of the negative effects of hyperglycaemia on trabecular bone quality that could lead to lower energy absorption and toughness and may explain the increased bone fragility in patients with T2D. Therefore, bone quality and increased bone fragility in patients with T2D cannot be explained by BMD alone [32]. In particular, traditional techniques for measuring bone fragility, such as DXA, do not perform well in patients with T2D and the Fracture Risk Assessment Tool (FRAX) usually underestimates fracture risk in this population [5]. However, new techniques for the assessment of trabecular microarchitecture in patients with T2D, such as TBS and high-resolution peripheral quantitative computed tomography (HR-pQCT), are emerging, although (HR-pQCT) involves significant costs and exposure to radiation [5]. Therefore, conflicting data may exist in relation to trabecular bone quality in patients with T2D depending on the applied assessment method [35]. For example, studies using standard HR-pQCT measures identified that trabecular microarchitecture was preserved in patients with T2D, while studies relying on other tools suggested that it may be impaired [35].

In line with our review, an earlier study by Patsch et al. [36] also found no significant differences in the microarchitectural parameters of the trabecular bone between patients with T2D for ≥10 years and controls [27]. Therefore, trabecular plate qualities, which suggest a normal or improved microstructure, may not explain the increased fracture risk in patients with inadequately controlled T2D [16,35]. Furthermore, the difference in the findings of this review with respect to TBS compared with some previous studies may be due to the fact participants in this review were community-dwelling volunteers and may have included fewer patients with severe illnesses, such as uncontrolled T2D [27].

In the present review, the incidence of hip and non-spine fracture did not differ significantly between patients with T2D and controls. In an earlier study by Wallander et al. [37], it was observed that only for individuals with T2D using insulin was fracture risk significantly increased.

Limitations

The small numbers of articles included in the systematic review and meta-analysis are limitations of this review. In addition, the number of articles included in the meta-analysis on some of the outcomes of interest was limited, and this would suggest that those results should be interpreted with caution. The high heterogeneity in some of the analyses conducted is also a limitation of this review, although sub-group analysis was conducted in the meta-analysis with large studies. The studies included in this review were granted ethical approval by the various ethics review boards and committees, except for Jawhar et al. [28] and Van Hulten et al. [30], both retrospective cohort studies that did not include any statements about the ethical approval of their studies.

## 5. Conclusions

The findings of this review revealed that T2D significantly increased (*p* < 0.05) lumbar spine, total hip, femoral neck, narrow neck BMD, and BMI compared with controls. However, T2D did not appear to have a significant effect (*p* > 0.05) on total BMD, areal BMD, TBS, trabecular microarchitectural parameters, and the incidence of hip and non-spine fractures compared to controls. We recommend that future research and clinical practice involving the assessment of bone quality in patients with T2D should not be limited only to BMD, but should include a variety of assessment tools, such as TBS and trabecular microarchitectural parameters.

## Figures and Tables

**Table 1:** Search Strategy.

Patient/Population	Outcomes	Study Design	Combining Search Terms
Type 2 diabetes or type 2 diabetes mellitus or t2dm	Bone mineral density or BMD or trabecular score or bone quality or fracture risk assessment	Cohort study or longitudinal study or observational study	Column 1 and Column 2 and Column 3

**Table 2:** The description and characteristics of included studies.

Citation/Country of Study and Year	Type of Study	Aim	Participants	Sample Size	Mean Age (Years)	Results/Findings
Bonds et al. [25] USA	Prospective cohort	To determine the risk of fracture in postmenopausal women with type 2 diabetes and determine whether risk varies by fracture site, ethnicity, and baseline bone density.	Postmenopausal women with T2D	Participants with T2D *n* = 5285 Control = *n* = 88,120	T2D 64.9 ± 7.0 Control = 63.5 ± 7.4	With fracture by location, women with diabetes had higher fracture rates at multiple sites (hip, pelvis, upper leg; lower leg, ankle, knee; foot; upper arm, shoulder, elbow; and spine and tailbone fractures (*p* < 0.0001)). The rate of fractures to the lower arm, wrist, and hand were equal in both groups. An elevated risk of fracture at multiple sites (hip, pelvis, upper leg; spine and tailbone) was reported among black women with diabetes (RR 1.33, 95% CI 1.00 –1.75) and compared with NHW women who have diabetes (RR 1.18, 95% CI 1.08–1.29).
Dennison et al. [22] UK	Hertfordshire Cohort	To explore whether the high bone density observed in Type 2 diabetes may be a result of the indirect effect of insulin resistance.	Men and women with T2D	Total *n* = 909 Men *n* = 465 Women *n* = 444	Men 64.8 ± 2.6 Women 66.4 ± 2.6 Men diagnosed as T2D *n* = 33 Women *n* = 32 diagnosed as T2D	Bone density was higher in newly diagnosed diabetic subjects, with relationships stronger in women (*p* < 0.001) than men (*p* < 0.05) and attenuated by adjustment for body mass index. In both sexes, positive correlations were observed between the total femur and femoral neck BMD with measures of insulin resistance (r = 0.17–0.22), with stronger results observed in women.
Heilmeier et al. [26] USA	Prospective cohort (Longitudinal cohort study)	To prospectively characterise the 5-year longitudinal changes in bone microarchitecture and strength in T2D postmenopausal women with and without a history of fragility fractures and to compare their changes to non-diabetic healthy postmenopausal controls using HR-pQCT.	Post-menopausal women with T2D	*n* = 32 women with T2D *n* = 10 Control *n* = 12 DMFx *n* = 10	DM 59.0 ± 4.1 Control 58.9 ± 5.5	The control group exhibited significant decreases in total BMD (−3.8%, Tt.BMD, *p* = 0.001) and Ct.Ar (−3.9% Ct.Ar, *p* = 0.007), and a significant increase in cortical pore diameter (Co: +8.8% Ct.Po.Dm, *p* = 0.007). In both the control and T2D groups there was a significant decrease in trabecular number (Tb.N) accompanied by a significant increase in trabecular thickness and trabecular spacing (control: +8.8% Tb.Th, *p* = 0.024, + 7.5% Tb.Sp, *p* = 0.017; DM: +8.4% Tb.Th, *p* = 0.039, 6.0% Tb.Sp, *p* = 0.032).
Iki et al. [27] Japan	Prospective cohort	To clarify associations between glycemic and insulin-resistance indices and TBS in community-dwelling elderly Japanese men, and whether pentosidine, an AGE, and bone turnover marker levels affect these associations.	Elderly Japanese men with T2D	Total *n* = 1683 Men with T2D *n* = 313 Control *n* = 1370	DM 72.8 ± 5.2 Control 72.9 ± 5.1	Men with T2D weighed significantly more and had a significantly higher aBMD compared to those without T2D. There was no significant difference in TBS and the frequency of past osteoporotic fractures between the two groups. Hyperglycaemia and elevated insulin resistance were associated with low TBS independently of bone turnover and pentosidine levels.
Jawhar et al. [28] United Arab Emirates (UAE)	Retrospective cohort	To assess the association between osteoporosis and T2D in females, with an emphasis on the identification of the major characteristics of BMD, T-score, and Z-score in female diabetic patients.	Women with T2D	Total *n* = 568 Pre- and postmenopausal women with diabetes *n* =141 Control *n* = 428	T2D 63.55 ± 9.15 Control 58.88 ± 11.71	The prevalence of osteoporosis was significantly higher (*p* ≤ 0.01) in the diabetic group. Younger diabetic patients (40–49 years) had significantly higher values (*p* ≤ 0.05) of BMD, T-score, and Z-score in the left femur and total hip. Diabetic patients in the age range of 50–59 years have significantly higher values of BMD and Z-score in the L3 region of the spine than other groups. Women who are obese have a significantly higher (*p* ≤ 0.001) BMD than non-obese women in the diabetes and control groups.
Mitama et al. [23] Japan	Cohort	To evaluate the combined effect of diabetes mellitus and one inflammatory marker (high-sensitive C-reactive protein [hs-CRP]) on the risk of incident fracture in a large-scale Japanese cohort.	Men and women with T2D	Men with T2D *n* = 413 Control *n* = 2110 Women with T2D *n* = 379 Control *n* = 3005	Men with T2D 68.2 ± 6.4 Control 67.6 ± 6.7 Women with T2D 7.3 ± 1.2 Control 68.0 ± 7.6	For both men and women, ageing, low BMD, previous fractures, and high CRP had significant associations with fracture. Fracture risk was significantly higher among the diabetes mellitus with high CRP group compared with the non-diabetes mellitus with low CRP group.
Oei et al. [16] The Netherlands	Prospective population-based cohort	To investigate if the intricate relationships between BMD, bone geometry, and fractures in type 2 diabetes are influenced by glucose control.	T2D	Total *n* = 4135 Non-diabetic (ND), adequately controlled diabetes (ACD) Inadequately controlled diabetes (ICD) (ICD) *n* = 217 Control (ACD) *n* = 203 (ND *n* = 3715)	ICD 68.5 ± 7.8 ACD Control 71.9 ± 7.6	Those with diabetes were older, had higher BMI, serum insulin, and creatinine levels, and used diuretics more frequently than those in the non-diabetes group. Participants with ICD had a higher fracture risk than individuals without diabetes. Poor glycaemic control in T2D is associated with fracture risk, high BMD, and thicker femoral cortices in narrower bones.
Pritchard et al. [29] Canada	Prospective cohort	To compare two-year changes in trabecular bone microarchitecture in women with and without type 2 diabetes.	Postmenopausal women with T2D	Women with T2D at baseline *n* = 30 Control *n* = 30 At follow-up with T2D *n* = 15 Control *n* = 22	Baseline women with T2D 71.1 ± 4.8 Control 70.7 ± 4.9 T2D 73.9 ± 3.6 Control 72.5 ± 4.9	At both the baseline and follow-up points, lumbar spine BMD was greater (*p* < 0.05) for women with diabetes than without diabetes. There were no differences in the change in other trabecular bone microarchitecture variables between groups.
Wang et al. [24] China	Prospective cohort	To investigate the association of vBMD and fasting plasma glucose in a large cohort of Chinese subjects and compare the vBMD in healthy and diabetic subjects. To compare the relationships between aBMD, vBMD, glucose, and fat mass in a subset of a Chinese cohort.	Men and women with diabetes	Total Men, *n* = 593 Women *n* = 444 Men with T2D *n* = 80 Control *n* = 513 Women with T2D *n* = 32 Control *n* = 412	Men with T2D 54.7 ± 10.3 Control 49.9 ± 9.6 Women with T2D 61.2 ± 9.7 Control 52.0 ± 9.9	Both men and women with diabetes were significantly older (*p* < 0.001). Both had a higher BMI than the non-diabetes women. Both women and men with diabetes had a lower vBMD compared to non-diabetic subjects, but this was non-significant after adjusting for age. In the DXA sub-cohort, aBMD was significantly higher in men with diabetes after adjusting for age.
Van Hulten et al. [30] Denmark	Retrospective cohort	To study the association between femoral neck (FN) bone mineral density (BMD), T-score, and fracture risk in individuals with and without type 2 diabetes (T2D).	Men and women with T2D	Total: Men *n* = 7069 Men with T2D *n* = 758 Control *n* = 6311 Women *n* = 35,129 Women with T2D *n* = 2362 Control *n* = 32,767	Men with T2D 68.5 ± SD 10.6 Control 65.4 ± 13.0 Women with T2D 70.0 ± 10.6 Control 65.5 ± 12.1	Crude IRs for hip fractures, non-spine fractures, and MOFs were not significantly different in women (age > 30) with T2D not using insulin (IR hip: 8.7; 95% CI 6.8–11.0) or using insulin (IR hip: 11.1; 95% CI 6.8–18.0) compared with women without T2D (IR hip: 7.0; 95% CI 6.6–7.4). For all three fracture types, IRs were not significantly different in men with T2D not using insulin (IR hip: 4.6; 95% CI 2.6–8.0) or using insulin (IR hip: 11.5; 95% CI 6.2–21.4) compared with men without T2D (IR hip: 6.3; 95% CI 5.5–7.1)

Abbreviations: adequately controlled diabetes (ACD); areal bone mineral density (aBMD); body mass index (BMI); bone mineral density (BMD); C-reactive protein (CRP); confidence interval (CI); control (Co); cortical area (Ct.Ar); cortical pore diameter (Ct.Po.Dm); dual-energy X-ray absorptiometry scan (DXA scan); femoral neck (FN); high inflammatory marker (hs-CRP); high-resolution peripheral quantitative computed tomography (HR-pQCT); inadequately controlled diabetes (ICD); incidence rates (IRs); major osteoporotic fracture (MOF); standard deviation (SD); non-diabetic (ND); non-Hispanic white (NHW); relative risk (RR); T2D postmenopausal women with a positive history of fragility fractures (DMFx); total bone mineral density (Tt.BMD); trabecular bone score (TBS); trabecular number (Tb.N); trabecular separation (Tb.Sp); trabecular thickness (Tb.Th); type 2 diabetes (T2D); volumetric bone mineral density (vBMD).

**Table 3:** Potential Confounding Variables of Included Studies.

Citation/Country of Study and Year	Duration of Diabetes (Yrs)	Glycaemic Control Measures	Potential Confounding Medications
Bonds et al. [25] USA	9.3 ± 10.0	Insulin usage (16.7%)	Some of the participants included in the study were on: Vitamin D; Oestrogen; Bisphosphonates; Steroids; Thiazide diuretics; Statins; Thyroid hormones.
Dennison et al. [22] UK	Not Reported	Not Reported	Participants on medications that alter bone metabolism (such as bisphosphonates) were excluded from the study, while women on hormone replacement therapy were included.
Heilmeier et al. [26] USA	64.4 ± 4.2	Not Reported	Exclusion criteria included the chronic (>6 months) use of bone-affecting medications (the intake of oestrogens, adrenal or anabolic steroids, antacids, anticoagulants, anticonvulsants, pharmacological doses of Vitamin A, fluorides, bisphosphonates, calcitonin, tamoxifen, parathyroid hormone [PTH], or thiazolidinediones).
Iki et al. [27] Japan	A median duration of disease of 10.5 years	Thiazolidinediones and other anti-diabetic drugs	Participants on medications known to affect bone metabolism (such as medications for uncontrolled hyperthyroid disease, parathyroid disease, type 1 diabetes, connective tissue disease, gastrectomy due to cancer or ulcer, prostate cancer with anti-androgen therapy, oral glucocorticoid therapy at any dose, bisphosphonate therapy for >6 months, and activated vitamin D use for >2 years) were excluded from the study.
Jawhar et al. [28] United Arab Emirates (UAE)	Not Reported	Oral antidiabetic medications (Not specified)	Not Reported
Mitama et al. [23] Japan	Not Reported	Not Reported	Participants who were under treatment for osteoporosis, rheumatoid arthritis, collagen diseases, and other inflammatory diseases were excluded from the study.
Oei et al. [16] The Netherlands	Not Reported	Insulin and antidiabetic medications (not specified)	Information on medication use included the use of antidiabetic medication, diuretics, hormonal replacement therapy, and systemic corticosteroids.
Pritchard et al. [29] Canada	≥5 years; At follow-up, women with type 2 diabetes had a diagnosis of diabetes for 18.8 ± 9.7 years.	At follow-up, the majority of participants (12/15 [80.0%]) were taking insulin or insulin in combination with another glucose-lowering intervention. The remaining participants were either taking metformin (2/15 [13.3%]) or no medication (1/15 [6.7%]).	Participants who were taking, or had taken in the past 24 months, any medication known to affect bone, including hormone therapy, calcitonin, selective oestrogen receptor modulator, parathyroid hormone, or bisphosphonate, or were taking oral glucocorticoids (≥2.5 mg/day for ≥3 months) were excluded from the study.
Wang et al. [24] China	Not Reported	Information on antidiabetic medication was restricted to insulin and/or oral antidiabetic medications or no medication use.	Not Reported
Van Hulten et al. [30] Denmark	6.1 ± 4.8	Glucagon-like peptide-1 receptor agonists Insulin Sodium glucose cotransporter 2 inhibitor Sulphonylurea Dipeptidyl-peptidase 4 inhibitor Thiazolidinediones Non-insulin antidiabetic drug	The use of the following medications in the 6 months before the index date was considered a potential cofounder: codeine, opioids, antidepressants, nitrates, loop diuretics, antipsychotics, anti-Parkinson medication, hormone replacement therapy, histamine type-2 receptor antagonists and proton pump inhibitors, anticonvulsants, statins, anti-osteoporotic medication, and corticosteroids.

**Table 4:** Results of the meta-analysis of the effect of Type 2 diabetes on bone quality.

		Patients with Type 2 Diabetes				
Outcomes	Number of Studies or Gender	Number of Participants	Statistical Method	Weighted Difference (95% CI)	*p*-Value	I^2^ %
Trabecular Bone Mineral Density	1	22	Mean Difference	2.04 [−1.28, 5.36]	*p* = 0.23	
Areal Bone Mineral Density	2	1037	Mean Difference	−0.01 [−0.04, 0.02]	*p* = 0.51	95
Total Bone Mineral Density	1	22	Mean Difference	5.33 [−1.20, 11.86]	*p* = 0.11	
Incidence of Non-Spine Fracture	2	6731	Mean Difference	−0.31 [−3.97, 3.36]	*p* = 0.87	78
Hole Size	1	35	Mean Difference	−0.04 [−0.31, 0.23]	*p* = 0.77	
Number of Holes	1	35	Mean Difference	1.00 [−9.28, 11.28]	*p* = 0.85	
Bone Volume Fraction	1	35	Mean Difference	0.10 [−0.50, 0.70]	*p* = 0.74	

## Supplementary Materials

The following supporting information can be downloaded at: https://www.mdpi.com/article/10.3390/ijerph22060910/s1, Table S1: PRISMA 2020 Checklist.

## References

[1] Ojo O., Kalocsányiová E., McCrone P., Elliott H., Milligan W., Gkaintatzi E. (2024). Non-Pharmacological Interventions for Type 2 Diabetes in People Living with Severe Mental Illness: Results of a Systematic Review and Meta-Analysis. Int. J. Environ. Res. Public Health.

[2] Cirovic A., Vujacic M., Petrovic B., Cirovic A., Zivkovic V., Nikolic S., Djonic D., Bascarevic Z., Djuric M., Milovanovic P. (2022). Vascular Complications in Individuals with Type 2 Diabetes Mellitus Additionally Increase the Risk of Femoral Neck Fractures Due to Deteriorated Trabecular Microarchitecture. Calcif. Tissue Int..

[3] Ojo O., Boateng J., Pacella R., Hanrahan A., Essex R., Dibley L. (2024). Factors Influencing the Care and Management of Diabetic Foot Ulcers: A Scoping Review. Endocr. Pract..

[4] Yang J., Zhang Y., Liu X., Chen B., Lei L. (2024). Effect of type 2 diabetes on biochemical markers of bone metabolism: A meta-analysis. Front. Physiol..

[5] Martínez-Montoro J.I., García-Fontana B., García-Fontana C., Muñoz-Torres M. (2022). Evaluation of Quality and Bone Microstructure Alterations in Patients with Type 2 Diabetes: A Narrative Review. J. Clin. Med..

[6] Sheu A., Blank R.D., Tran T., Bliuc D., Greenfield J.R., White C.P., Center J.R. (2023). Associations of Type 2 Diabetes, Body Composition, and Insulin Resistance with Bone Parameters: The Dubbo Osteoporosis Epidemiology Study. JBMR Plus.

[7] Donnelly E. (2011). Methods for assessing bone quality: A review. Clin. Orthop. Relat. Res..

[8] Morgan E.F., Unnikrisnan G.U., Hussein A.I. (2018). Bone Mechanical Properties in Healthy and Diseased States. Annu. Rev. Biomed. Eng..

[9] Napoli N., Incalzi R.A., De Gennaro G., Marcocci C., Marfella R., Papalia R., Purrello F., Ruggiero C., Tarantino U., Tramontana F. (2021). Bone fragility in patients with diabetes mellitus: A consensus statement from the working group of the Italian Diabetes Society (SID), Italian Society of Endocrinology (SIE), Italian Society of Gerontology and Geriatrics (SIGG), Italian Society of Orthopaedics and Traumatology (SIOT). Nutr. Metab. Cardiovasc. Dis..

[10] Weber D.R., Long F., Zemel B.S., Kindler J.M. (2022). Glycemic Control and Bone in Diabetes. Curr. Osteoporos. Rep..

[11] Misof B.M., Blouin S., Andrade V.F.C., Roschger P., Borba V.Z.C., Hartmann M.A., Zwerina J., Recker R.R., Moreira C.A. (2022). No evidence of mineralization abnormalities in iliac bone of premenopausal women with type 2 diabetes mellitus. J. Musculoskelet. Neuronal Interact..

[12] Ma L., Oei L., Jiang L., Estrada K., Chen H., Wang Z., Yu Q., Zillikens M.C., Gao X., Rivadeneira F. (2012). Association between bone mineral density and type 2 diabetes mellitus: A meta-analysis of observational studies. Eur. J. Epidemiol..

[13] Schwartz A.V. (2016). Epidemiology of fractures in type 2 diabetes. Bone.

[14] Wang C., Liu J., Xiao L., Liu D., Yan W., Hu T., Li K., Hua X., Zeng X. (2020). Comparison of FRAX in postmenopausal Asian women with and without type 2 diabetes mellitus: A retrospective observational study. J. Int. Med. Res..

[15] Sheu A., White C.P., Center J.R. (2024). Bone metabolism in diabetes: A clinician’s guide to understanding the bone-glucose interplay. Diabetologia.

[16] Oei L., Zillikens M.C., Dehghan A., Buitendijk G.H.S., Castaño-Betancourt M.C., Estrada K., Stolk L., Oei E.H.G., van Meurs J.B.J., Janssen J.A.M.J.L. (2013). High bone mineral density and fracture risk in type 2 diabetes as skeletal complications of inadequate glucose control: The Rotterdam Study. Diabetes Care.

[17] Trandafir A.-I., Sima O.-C., Gheorghe A.-M., Ciuche A., Cucu A.-P., Nistor C., Carsote M. (2023). Trabecular Bone Score (TBS) in Individuals with Type 2 Diabetes Mellitus: An Updated Review. J. Clin. Med..

[18] Page M.J., McKenzie J.E., Bossuyt P.M., Boutron I., Hoffmann T.C., Mulrow C.D., Shamseer L., Tetzlaff J.M., Akl E.A., Brennan S.E. (2021). The PRISMA 2020 statement: An updated guideline for reporting systematic reviews. BMJ.

[19] Critical Appraisal Skills Programme (2018). CASP Cohort Study Checklist. https://casp-uk.net/casp-tools-checklists/cohort-study-checklist/.

[20] The Nordic Cochrane Centre (2014). Review Manager (RevMan) [Computer Program].

[21] Higgins J.P.T., Green S. (2009). Cochrane Handbook for Systematic Reviews of Interventions.

[22] Dennison E.M., Syddall H.E., Aihie Sayer A., Craighead S., Phillips D.I.W., Cooper C. (2004). Type 2 diabetes mellitus is associated with increased axial bone density in men and women from the Hertfordshire Cohort Study: Evidence for an indirect effect of insulin resistance?. Diabetologia.

[23] Mitama Y., Fujiwara S., Yoneda M., Kira S., Kohno N. (2017). Association of type 2 diabetes and an inflammatory marker with incident bone fracture among a Japanese cohort. J. Diabetes Investig..

[24] Wang L., Zhao K., Zha X., Ran L., Su H., Yang Y., Shuang Q., Liu Y., Xu L., Blake G.M. (2022). Hyperglycemia Is Not Associated With Higher Volumetric BMD in a Chinese Health Check-up Cohort. Front. Endocrinol..

[25] Bonds D.E., Larson J.C., Schwartz A.V., Strotmeyer E.S., Robbins J., Rodriguez B.L., Johnson K.C., Margolis K.L. (2006). Risk of fracture in women with type 2 diabetes: The Women’s Health Initiative Observational Study. J. Clin. Endocrinol. Metab..

[26] Heilmeier U., Joseph G.B., Pasco C., Dinh N., Torabi S., Darakananda K., Youm J., Carballido-Gamio J., Burghardt A.J., Link T.M. (2021). Longitudinal Evolution of Bone Microarchitecture and Bone Strength in Type 2 Diabetic Postmenopausal Women With and Without History of Fragility Fractures-A 5-Year Follow-Up Study Using High Resolution Peripheral Quantitative Computed Tomography. Front. Endocrinol..

[27] Iki M., Fujita Y., Kouda K., Yura A., Tachiki T., Tamaki J., Winzenrieth R., Sato Y., Moon J.-S., Okamoto N. (2017). Hyperglycemia is associated with increased bone mineral density and decreased trabecular bone score in elderly Japanese men: The Fujiwara-kyo osteoporosis risk in men (FORMEN) study. Bone.

[28] Jawhar D.S., Hassan N.A., Shamssain M.H. (2020). Dual-energy x-ray absorptiometry scan (DXA) findings in diabetic and non-diabetic female: A retrospective cohort study. Med. J. Malays..

[29] Pritchard J.M., Giangregorio L.M., Atkinson S.A., Beattie K.A., Inglis D., Ioannidis G., Gerstein H., Punthakee Z., Adachi J.D., Papaioannou A. (2013). Changes in trabecular bone microarchitecture in postmenopausal women with and without type 2 diabetes: A two year longitudinal study. BMC Musculoskelet. Disord..

[30] Van Hulten V., Driessen J.H.M., Andersen S., Kvist A., Viggers R., Bliuc D., Center J.R., Brouwers M.C.J.G., Vestergaard P., van den Bergh J.P. (2024). Fracture risk revisited: Bone mineral density T-score and fracture risk in type 2 diabetes. Diabetes Obes. Metab..

[31] Sosa M., Saavedra P., Jódar E., Lozano-Tonkin C., Quesada J.M., Torrijos A., Pérez-Cano R., Nogués X., Díaz-Curiel M., Moro M.J. (2009). Bone mineral density and risk of fractures in aging, obese post-menopausal women with type 2 diabetes. The GIUMO Study. Aging Clin. Exp. Res..

[32] Sihota P., Yadav R.N., Dhaliwal R., Bose J.C., Dhiman V., Neradi D., Karn S., Sharma S., Aggarwal S., Goni V.G. (2021). Investigation of Mechanical, Material, and Compositional Determinants of Human Trabecular Bone Quality in Type 2 Diabetes. J. Clin. Endocrinol. Metab..

[33] La Fontaine J., Shibuya N., Sampson H.W., Valderrama P. (2011). Trabecular quality and cellular characteristics of normal, diabetic, and charcot bone. J. Foot Ankle Surg..

[34] Li T., Hu L., Yin X.-L., Zou Y., Fu H.-Y., Li H.-L. (2022). Prevalence and Risk Factors of Osteoporosis in Patients with Type 2 Diabetes Mellitus in Nanchang (China): A Retrospective Cohort Study. Diabetes Metab. Syndr. Obes. Targets Ther..

[35] Starr J.F., Bandeira L.C., Agarwal S., Shah A.M., Nishiyama K.K., Hu Y., McMahon D.J., Guo X.E., Silverberg S.J., Rubin M.R. (2018). Robust Trabecular Microstructure in Type 2 Diabetes Revealed by Individual Trabecula Segmentation Analysis of HR-pQCT Images. J. Bone Miner. Res..

[36] Patsch J.M., Burghardt A.J., Yap S.P., Baum T., Schwartz A.V., Joseph G.B., Link T.M. (2013). Increased cortical porosity in type 2 diabetic postmenopausal women with fragility fractures. J. Bone Miner. Res..

[37] Wallander M., Axelsson K.F., Nilsson A.G., Lundh D., Lorentzon M. (2016). Type 2 diabetes and risk of hip fractures and non-skeletal fall injuries in the elderly: A study from the fractures and fall injuries in the elderly cohort (FRAILCO). J. Bone Miner. Res..

